# *In vitro* co-cultures of human gut bacterial species as predicted from co-occurrence network analysis

**DOI:** 10.1371/journal.pone.0195161

**Published:** 2018-03-30

**Authors:** Promi Das, Boyang Ji, Petia Kovatcheva-Datchary, Fredrik Bäckhed, Jens Nielsen

**Affiliations:** 1 Department of Biology and Biological Engineering, Chalmers University of Technology, Gothenburg, SE, Sweden; 2 Wallenberg Laboratory, Department of Molecular and Clinical Medicine, University of Gothenburg, Gothenburg, SE, Sweden; 3 Novo Nordisk Foundation Center for Basic Metabolic Research, Section for Metabolic Receptology and Enteroendocrinology, Faculty of Health Sciences, University of Copenhagen, Copenhagen, Denmark; 4 Novo Nordisk Foundation Center for Biosustainability, Technical University of Denmark, DK Lyngby, Denmark; Virginia Commonwealth University, UNITED STATES

## Abstract

Network analysis of large metagenomic datasets generated by current sequencing technologies can reveal significant co-occurrence patterns between microbial species of a biological community. These patterns can be analyzed in terms of pairwise combinations between all species comprising a community. Here, we construct a co-occurrence network for abundant microbial species encompassing the three dominant phyla found in human gut. This was followed by an *in vitro* evaluation of the predicted microbe-microbe co-occurrences, where we chose species pairs *Bifidobacterium adolescentis* and *Bacteroides thetaiotaomicron*, as well as *Faecalibacterium prausnitzii* and *Roseburia inulinivorans* as model organisms for our study. We then delineate the outcome of the co-cultures when equal distributions of resources were provided. The growth behavior of the co-culture was found to be dependent on the types of microbial species present, their specific metabolic activities, and resulting changes in the culture environment. Through this reductionist approach and using novel *in vitro* combinations of microbial species under anaerobic conditions, the results of this work will aid in the understanding and design of synthetic community formulations.

## Introduction

The nature of microbe-microbe interaction ranges from antagonism to mutualism, and determines the overall composition and function of a microbial community [1]. For instance, one might envisage that antagonistic microbes or those that compete each other for the same niche may negatively correlate. In contrast, microbes with mutualistic relationships such as cooperation or mutualism may positively correlate across samples. However, in reality, based on the environmental preferences, microorganisms can coexist with highly diverse patterns in different locations with indirect reasons. In addition to numerous factors, ecological interactions between microbial species in a community and their enzymatic potential to consume available carbon sources modulates the composition of gut microbiota to a large extent [2,3]. Typically, dietary carbohydrates are a major source of carbon and energy for the gut microbiota [4]. The end products from these fermentations are short-chain fatty acids (SCFAs), known to impart several physiological effects on human health [5].

Advancement in sequencing technologies has generated an abundance of data on the composition of microbes that live in and on human body [6]. Typically, metagenomic studies have either described the structure of microbial communities with a focus on the total number of phyla or lineages found in a single sample [7], or have compared the relative abundance of every phylum between microbial communities [8]. The development of tools such as co-occurrence network analysis now enables the analysis of high-dimensional and complex distributions of metagenomics data [9–11]. Furthermore, through careful experimental design, co-occurrence patterns between microbes across diverse communities can be verified, which can provide important insights into the structural properties of the gut ecosystem. Although there are few studies that have used metagenomics data to explore the correlation between the abundance of microbial pairs, there is insufficient evidence to test the network prediction between dominant bacterial species from human gut samples under *in vitro* conditions.

In order to determine statistically significant co-occurrence patterns, sufficiently large sample sizes are needed. We therefore used a gut microbial gene catalogue, which was constructed from four different studies conducted over three continents [6,7,12–14]. To investigate our *in-silico* predictions from the resulting co-occurrence network, we co-cultured i) *Bifidobacterium adolescentis* from the Actinobacteria phylum, together with *Bacteroides thetaiotaomicron* from Bacteroidetes, both of which are known polysaccharide degraders; ii) *Faecalibacterium prausnitzii* together with *Roseburia inulinivorans*, both from the Firmicutes phylum and well-known butyrate producers in the human gut [4,15]. We chose these bacterial species pair for the following reasons. Co-culture of Bifidobacterium and Bacteroides have not been studied yet when grown on a mixture of carbon sources, and no study have shown the influence of acetate on the joint behavior of Faecalibacterium and Roseburia in any *in vitro* growth medium. These four species have gained interest as several human gut metagenomics studies have shown that their abundance is associated with healthy gut microbiome, maybe because their genetic flexibility allows them to survive in various ecological niches. Therefore, data on these species would contribute to our understanding of human gut microbiome as they also belong to the core microbiome [16]. In particular, *Bacteroides thetaiotaomicron* is of great importance in terms of symbiotic bacteria-host relationships within the human intestine, as well as for its potential to break down plant polysaccharides [17]. It also contributes to the post-natal gut development and host physiology[18]. *Bifidobacterium adolescentis* is a key microbe of the adult-associated Bifidobacteria [19]. Apart from its potential therapeutic use as a probiotic, this bacterial species has also been found to have a potential impact on the response to cancer treatment [20].

## Experimental methods

### Co-occurrence network analysis

782 human gut shotgun metagenomes from four different studies, Sweden [14], MetaHIT [13], HMP [6] and China [7] have been extensively analyzed by MEDUSA in our previous study [12]. The species count table was downloaded from the MEDUSA website (http://www.metabolicatlas.com/medusa). Assuming the limitations of available correlation analysis methods, we chose two suitable methods with respect to our dataset. To generate co-occurrence networks, we employed and compared two known methods: i) Spearman correlation [21], which has been used for non-parametric statistical testing to measure correlation and ii) SparCC method [22] which has been known to offer high-precision detection of linear relationships in a compositional dataset [23]. Spearman correlation coefficients were calculated from the relative species abundance data across individuals, and multiple comparisons were corrected with the Bonferroni method [24]. The thickness of the edges represents the level of association depending on the value of Spearman’s correlation coefficient. All the statistical analyses were performed in R. To reduce the dimensionality of the statistical analyses, we identified significantly correlated species pairs and then subsequently clustered abundance of associated species using a straightforward hierarchical clustering algorithm. An edge was assigned between two species if the absolute correlation coefficient was greater than or equal to 0.4, with an adjusted p-value less than 0.01. SparCC was run with default parameters and 100 bootstraps where significance of the network was filtered by significant p values less than 0.01 and absolute value of correlation score greater than or equal to 0.3. Both the networks were subsequently visualized using igraph with LGL layout [25]. LGL applies a force-directed iterative layout guided by a minimal spanning tree of the network in order to generate coordinates for the vertices in two or three dimensions.

### Bacterial strains

*Bifidobacterium adolescentis* L2-32, *Faecalibacterium prausnitzii* A2-165 and *Roseburia inulinivorans* A2-194 (DSM 16841) were kindly provided by Dr. Karen Scott (The Rowett Institute of Nutrition and Health, Aberdeen, UK). *Bacteroides thetaiotaomicron* 29148 was ordered from ATCC. All bacterial strains were maintained at 37°C in Hungate tubes (Ochs Laborbedarf, Germany) under oxygen-free CO_2_ in yeast extract, casitone and fatty acid (YCFA) medium [26] and cultured under strict anaerobic conditions in an anaerobic chamber (Coy Lab Products, Grass Lake, MI, USA).

### Media preparation

The composition of the base medium per 100 ml, is as follows: 0.25 g yeast extract (BD), 1.0 g casitone (BD), 0.4 g NaHCO_3_ (Merck), 0.045 g K_2_HPO_4_ (Merck), 0.045 g KH_2_PO_4_ (Merck), 0.09 g NaCl (Merck), 0.09 g (NH_4_)_2_SO_4_ (Merck), 0.009 g MgSO_4_ · 7H_2_O (Merck), 0.009 g CaCl_2_ (Merck), 0.1 mg resazurin (Sigma-Aldrich), and 1 mg hemin (Sigma-Aldrich). In addition, the final concentrations of following short-chain fatty acids (SCFA) were included (final concentrations): acetate (33 mM); propionate (9 mM); isobutyrate, isovalerate, and valerate (1 mM each). Finally, the volume was adjusted with dH_2_O in a conical Erlenmeyer flask. The medium was boiled in a microwave to dissolve the complex compounds. After the medium cools down, it was supplemented with cysteine (0.1 g), followed by boiling under oxygen-free CO_2_ atmosphere for 3–4 minutes. After autoclaving at 120°C for 15 min, filter sterilized solutions of vitamins (1 μg biotin, 1 μg cobalamin, 3 μg p-aminobenzoic acid, 5 μg folic acid, 15 μg pyridoxamine, 5 μg thiamine and 5 μg riboflavin) per 100 ml of medium. All SCFAs and vitamins were purchased from Sigma-Aldrich. The final pH of medium was maintained to 7.2 ± 0.1.

In YCGMS medium, carbon sources such as glucose (G), maltose (M), cellobiose (C), and soluble starch (S) were added to a final concentration of 0.2% (wt/vol) of each. We selected starch as a substrate because it serves as the major source of carbon and energy for the gut microbiota [4,27,28]. To encourage the growth of the fastidious species, simple sugars were added to the medium. This media was used for the *B*. *adolescentis* L2-32 and *B*. *thetaiotaomicron* ATCC 29148 co-culture experiment. Short chain fatty acid (SCFA) mix was added to YCFA medium, but not to YCGMS medium, as they do not consume them. All other components were the same between the YCFA and YCGMS media.

For experiments with *F*. *prausnitzii* and *R*. *inulinivorans*, the bacteria were inoculated into a medium that contained yeast extract, casitone, glucose and disaccharide (maltose and cellobiose) with the addition of SCFA mix (YCFAGD medium) and without the addition of SCFA mix (YCGD medium). It has been found that acetate produced from other bacteria in a community enhance butyrate formation by *Faecalibacterium prausnitzii* through cross-feeding [29]. Hence, we used two growth media formulations, YCFAGD (with acetate) and YCGD (without acetate) to investigate these findings, which might influence their co-occurrence pattern.

### Culture growth conditions

Each individual bacterial species was sub-cultured on an agar plate with YCFA medium [26] in an anaerobic chamber. A single colony was transferred in a Hungate tube containing 7.5 ml of the YCFA medium and incubated overnight and used as an inoculum. The fermentations were performed in 100 ml serum bottles (Ochs Laborbedarf, Germany), which contained 50 ml of the respective autoclaved growth media and oxygen-free CO_2_ in the gas phase. Each bottle was inoculated with 2% (vol/vol) inoculum. The bacteria were allowed to grow by inoculating equal volumes of inoculum in both the mono- and co-culture respectively. Media without supplementation of inoculum were used as control. The bottles were incubated at 37°C for a maximum of 56 hours, and each experiment was performed in triplicates.

### Genomic DNA purification and quantitative PCR

Genomic DNA was isolated from a pellet, which was obtained after centrifuging 1 ml of microbial culture at 16 000 rpm for 5 min, using the NucleoSpin® Soil kit (MACHEREY-NAGEL, Germany). The manufacturer’s instructions were followed, with the exception that the vortex step was replaced with repeated bead beating twice at 5.0 m/s for 60 seconds using the FastPrep®-24 Instrument (MP Biomedicals), with 5 minutes of incubation on ice after each round of bead-beating.

To quantify the abundance of the two-bacterial species in co-culture, we employed 16S rRNA quantitative PCR using a CFX96 Real-Time System (Bio-Rad Laboratories). Samples were analyzed in a 25-μl reaction mix consisting of 12.5 μl 1xSYBR Green Master Mix buffer (Thermo Scientific, Waltham, Massachusetts, USA), 0.2 μM of each primer, and 5 μl of template DNA, with water as a control. qPCR was performed as reported previously: *B*. *adolescentis*, *R*. *inulinivorans* and *F*. *prausnitzii* [30] and for *B*. *thetaiotaomicron* [31]. Standard curves for quantification of the different bacteria consisted of ten-fold dilutions of purified 16S rRNA PCR product, in the range of 10^8^ to 10^0^ copies. Later, the obtained 16S rRNA values were normalized with respect to each species’ 16S rRNA copy number.

### Quantification of extracellular metabolites

One-millilitre of homogenized microbial cultures were concentrated by centrifugation (16 000 rpm, 5 min) and the supernatant was used for analysis. Glucose, disaccharides, acetate, butyrate, lactate, formate, succinate and propionate were measured by high pressure liquid chromatography (HPLC). For this analysis, the RI and UV detectors and an Aminex HPX-87H column (Biorad, Irvine, CA, USA) were used to separate and quantify all the metabolites in the supernatants of the cultures. The column was eluted with 5 mM H_2_SO_4_ at a flow rate of 0.6 ml/min at 65°C. To quantify the consumption of starch, 1 ml of bacterial cultures grown in YCGMS media were filtrated and a mild post-hydrolysis method were applied. H_2_SO_4_ (4% volume of solute/volume of solution) was used to depolymerize starch into its monomer constituents. The samples were incubated at 120°C for 30 minutes. Quantification of the metabolites was performed as previously reported [32].

### pH perturbation experiments

*B*. *thetaiotaomicron* was grown for 24 h in YCFA medium as described above. Around 12 h, the pH of the medium was lowered to pH 3.5 with 6 M HCl. Six hours later; the pH of the medium was increased to pH 8.5 with 5 M NaOH. In parallel to this, a similar set of experiments was performed in which the pH range and the order of pH adjustment were altered. Around 12 h, the pH of the medium was increased to pH 6.5 with 5 M NaOH. Six hours later, the pH of the medium was lowered to pH 5.5 with 6 M HCl. Culture media without pH change, and media without inoculum were used as controls. Equal volumes of inoculum were inoculated into each of the samples, except the cell-free control. Each set of experiments was performed in duplicates.

### Statistical methods

Results plotted are the mean values with error bars corresponding to the standard deviation of the biological replicates at each time-point. Statistical comparison of 16S rRNA abundance data between mono-culture and co-culture of each species in same or different medium was evaluated by Student’s t-test using GraphPad Prism version 7.00 for Mac, GraphPad Software, La Jolla California USA. Significance was set at p-value of less than 0.01 (two-tailed).

## Results

### Analysis of the co-occurrence network between abundant genera in the human gut

**Fig 1:**
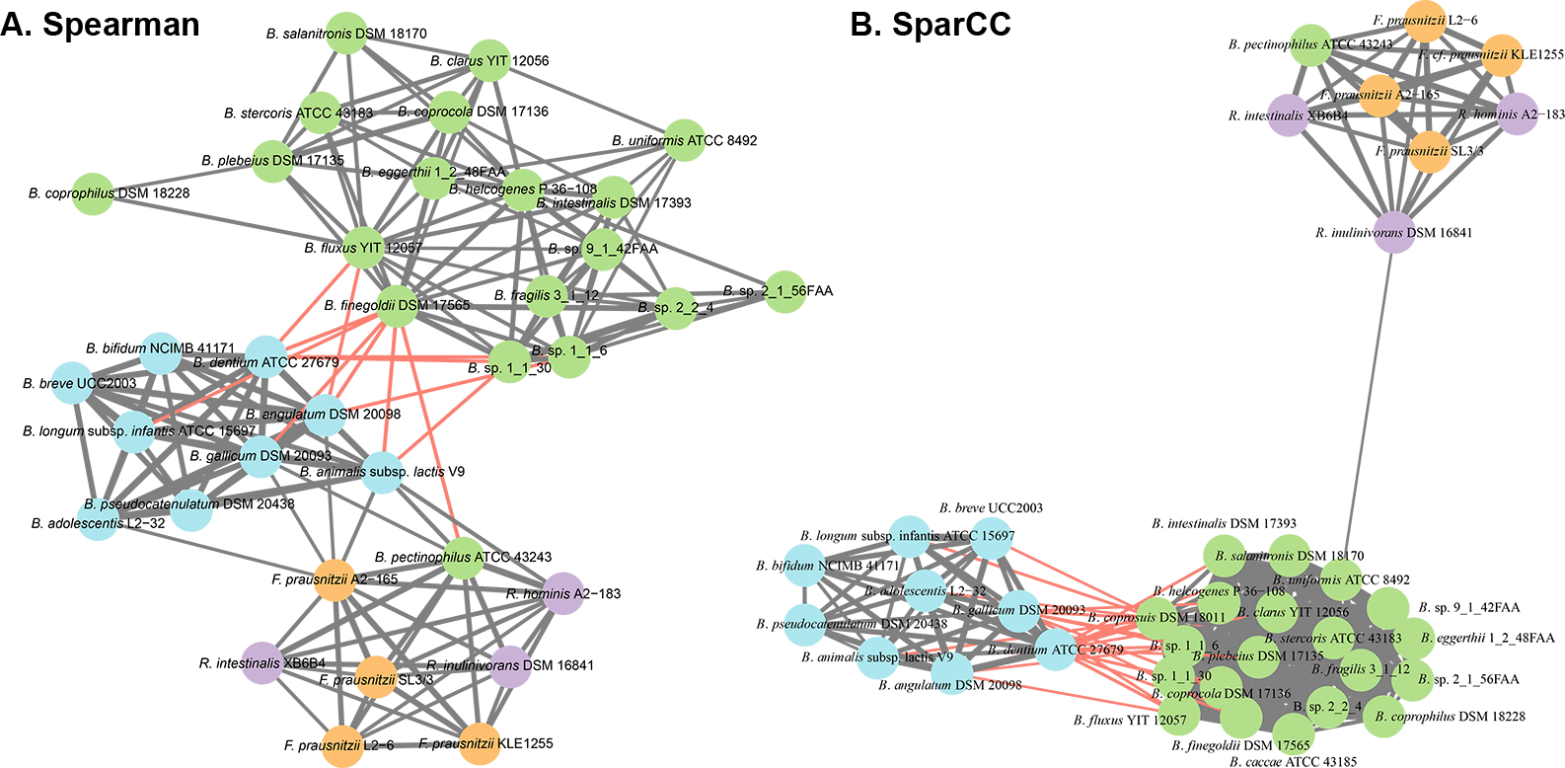
Co-occurrence network of significantly abundant species. A. Spearman correlation method. B. SparCC method. The nodes represent microbial species from the genus *Bacteroides* (green), *Bifidobacterium* (blue), *Faecalibacterium* (orange) and *Roseburia* (purple). The thickness of the edges represents the level of association depending on the value of Spearman’s correlation coefficient or the SparCC score respectively. Positive correlations are predicted to exist between species of Faecalibacterium and Roseburia, as shown in gray lines. Negative correlations are predicted to exist between species of Bacteroides and Bifidobacterium, as shown in red lines, except for (*Bacteroides pectinophilus*).

To identify the non-random co-occurrence patterns among species of abundant human gut genera, co-occurrence network analysis was performed. Both the methods produced similar numbers and types of significant edges for the same data. The resulting network showed that the abundance of species from the genera Bacteroides and Bifidobacterium were negatively correlated. *Bacteroides pectinophilus* ATCC 43243 was the only exception, as its abundance negatively correlated with other *Bacteroides* species, and positively correlated with Bifidobacteria (Fig 1). Interestingly, the strongest correlation was present between different species of the genus *Bifidobacterium*. Previous experimental studies complement this specific observation [33]. On the other hand, species from the genus *Faecalibacterium* co-occurred with species from the genus *Roseburia* (Fig 1).

Although these positive and negative correlations can describe the tendency of different species to co-occur among various conditions, it does not reveal the underlying cause of such patterns. Because co-occurrence networks are undirected weighted network, there is no directionality of the interactions. For example, two species exhibiting a significantly negative correlation in abundance could be directly interacting through nutrient competition, or they could differ in physiological requirements to such an extent that they never occupy the same niche. Likewise, a positive correlation may simply indicate a shared preference for a particular combination of environmental conditions, or could be a true ecological interaction where, e.g., two species can grow better through metabolite exchanges. We, therefore, sought to experimentally investigate pairs of species from our co-occurrence network analysis that were found to exhibit positive and negative correlations, to better understand the potential causes of such behavior.

### *In-vitro* culture analysis to study the co-occurrence pattern between *Bifidobacterium adolescentis* and *Bacteroides thetaiotaomicron*

**Fig 2:**
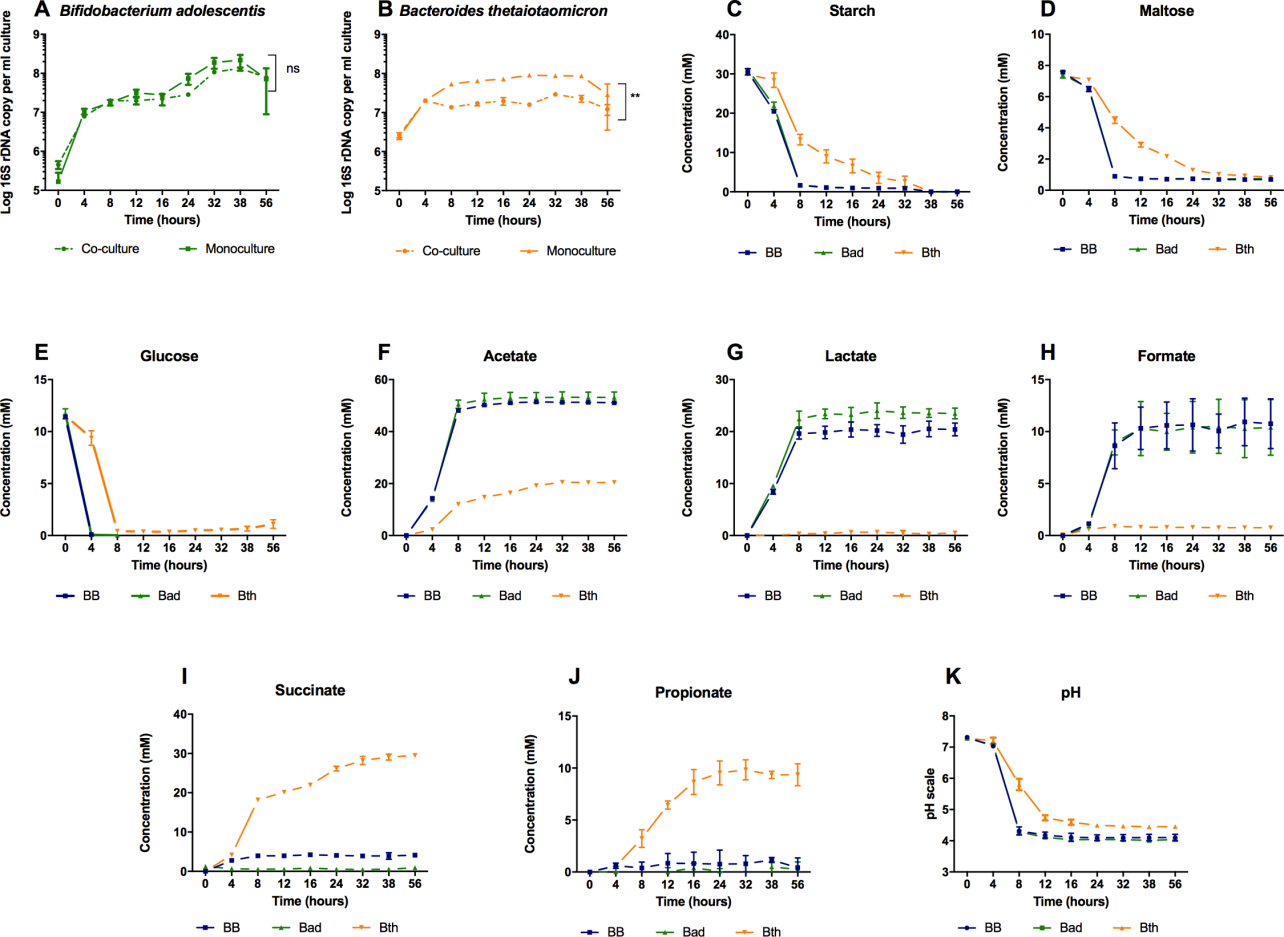
Profiles of log_10_ 16S rRNA copies, extracellular metabolite concentrations, and pH of *Bifidobacterium adolescentis* and *Bacteroides thetaiotaomicron* in mono- and co-cultures in YCGMS (Yeast Casitone glucose maltose starch) medium for 56 h. A and B. Log_10_ 16S rRNA copies per ml of culture, C-E. Starch, maltose and glucose consumption. F-H. Acetate, lactate and formate production. I-J. Succinate and propionate production. K. pH profile of mono- and co-cultures of *B*. *adolescentis* and *B*. *thetaiotaomicron*. BB = co-culture of *Bifidobacterium adolescentis* and *Bacteroides thetaiotaomicron*, Bad = mono-culture of *B*. *adolescentis*, Bth = mono-culture of *B*. *thetaiotaomicron*. Experiments were performed in triplicates and error bars represent the standard deviation between each biological replicate. P-values less than and greater than 0.01 are summarized with two asterisks and ‘non-significant (ns)’ respectively.

The co-occurrence network analysis showed that members of the genera *Bacteroides* and *Bifidobacterium* were generally negatively correlated, suggesting that species of these genera grown in co-culture may not grow as optimally as they would in mono-culture. To determine if, and through what mechanism(s), this was the case, we grew *Bifidobacterium adolescentis* and *Bacteroides thetaiotaomicron* in mono- and co-cultures *in vitro*. The pH profile and extracellular metabolite concentrations were measured to evaluate their fermentation activity. Our data showed that *B*. *adolescentis* exhibited no significant difference in abundance (Student’s t-test, p < 0.01) and fermentation activity in both the mono- and co-culture conditions (Fig 2A, 2C–2H). For *B*. *thetaiotaomicron*, however, the abundance of 16S rRNA gene copies was significantly different in the co-culture from the mono-culture experiment (Student’s t-test, p < 0.01). Growth of *B*. *thetaiotaomicron* reached saturation around 4 h and 8 h in the co-culture and mono-culture, respectively (Fig 2B). Furthermore, the metabolite consumption and production dynamics of *B*. *thetaiotaomicron* were notably different in the co-culture than the mono-culture (Fig 2C–2J). Glucose and maltose were depleted within 15 h of fermentation in all cultures (Fig 2D, 2E). In the mono-cultures, consistent with previous findings [2], *B*. *adolescentis* produced acetate, lactate and formate (Fig 2F–2H), whereas *B*. *thetaiotaomicron* produced acetate, succinate and propionate (Fig 2F and 2I–2J). In the co-culture, the metabolites produced in the greatest quantities were acetate, lactate and formate, and were at similar levels to those found in the *B*. *adolescentis* mono-culture. However, the levels of succinate and propionate in the co-culture were lower compared to the *B*. *thetaiotaomicron* mono-culture (Fig 2I–2J). The pH of the co-culture dropped dramatically from 7.2 to 4.0 after 8 h of fermentation, which is in agreement with the increased levels of acetate and lactate. The pH of the *B*. *thetaiotaomicron* mono-culture decreased at a slower rate, reaching only 5.8 at 8 h of fermentation (Fig 2K). This suggested that the lower pH achieved in the co-culture could potentially slow the growth and metabolism of *B*. *thetaiotaomicron* in the presence of *B*. *adolescentis*, after approximately 4 h of growth irrespective of available carbon substrate in the medium.

A separate follow-up experiment was performed to assess the ability of *B*. *thetaiotaomicron* to grow during pH perturbation. In agreement with previous reports, a decrease in pH limited the growth of *B*. *thetaiotaomicron* in the co-culture (S1 Fig) [34,35].

Collectively, our *in vitro* data suggests that one potential cause of the negative correlation identified between *B*. *adolescentis* and *B*. *thetaiotaomicron* in the network analysis could be the result of a negative interaction, whereby the metabolic products of *B*. *adolescentis* decrease the environmental pH enough to impart a growth-inhibitory effect on *B*. *thetaiotaomicron*.

### *In-vitro* culture analysis to study the co-occurrence pattern between *Faecalibacterium prausnitzii* and *Roseburia inulinivorans*

The co-occurrence network analysis showed that members of the genera Faecalibacterium and Roseburia were generally positively correlated, suggesting that species of these genera grown in co-culture are likely to grow as optimally than or as they would in mono-culture. To evaluate whether this was true, we grew *Faecalibacterium prausnitzii and Roseburia inulinivorans* in mono- and co-cultures *in vitro*.

**Fig 3:**
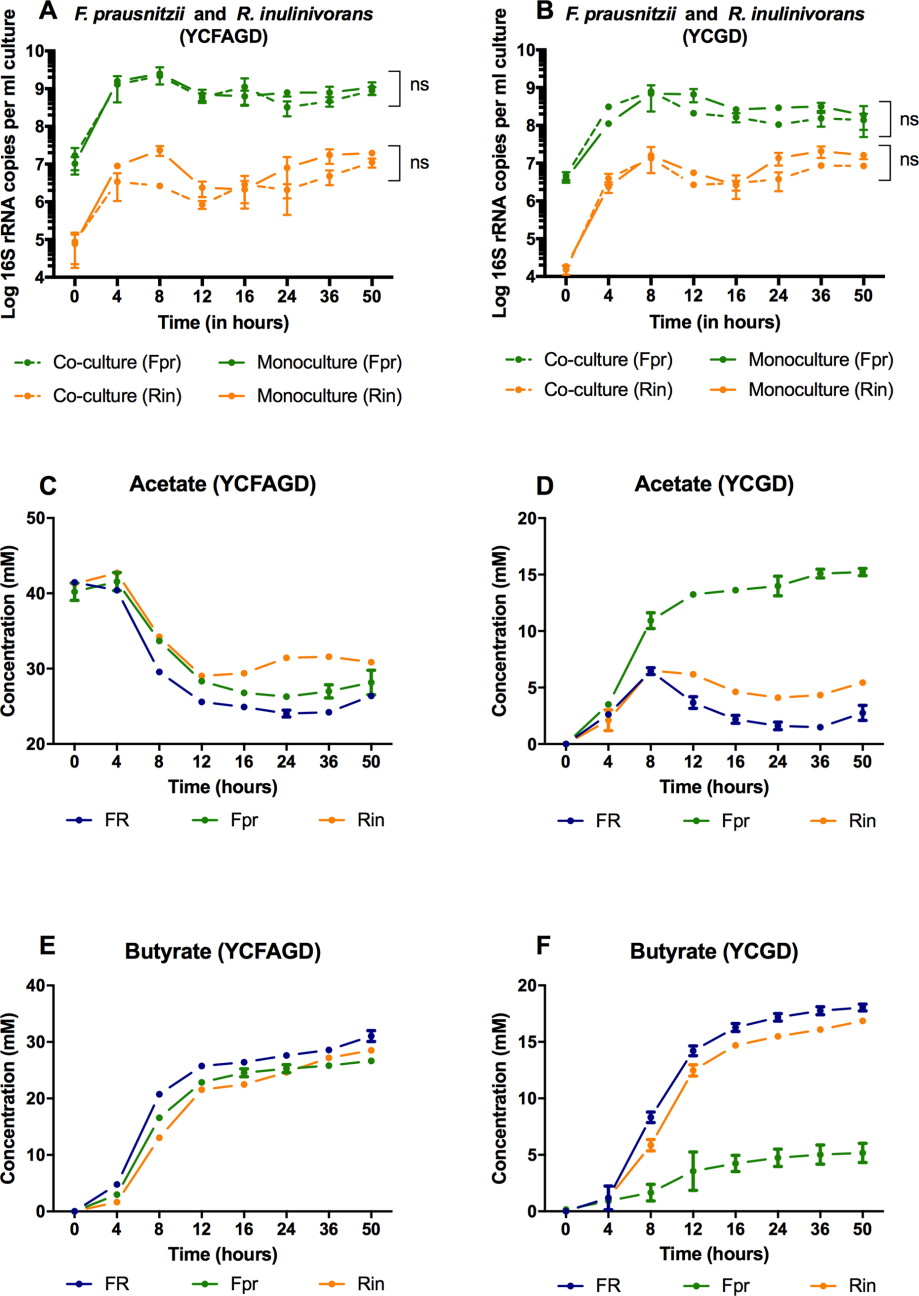
Profiles of log_10_ 16S rRNA copies and extracellular metabolite concentrations, of *Faecalibacterium prausnitzii* and *Roseburia inulinivorans* mono- and co-cultures in YCFAGD (Yeast Casitone free acetate glucose disaccharide) and YCGD (Yeast Casitone glucose disaccharide) medium for 50h. A-B Log_10_ 16S rRNA gene copies per ml culture, C-F. Acetate and butyrate concentration of *F*. *prausnitzii* and *R*. *inulinivorans* mono- and co-cultures in YCFAGD and YCGD medium. FR = co-culture of *Faecalibacterium prausnitzii* and *Roseburia inulinivorans*, Fpr = mono-culture of *Faecalibacterium prausnitzii*, Rin = mono-culture of *Roseburia inulinivorans*. Experiments were performed in triplicates and error bars represent the standard deviation between each biological replicate. P-values less than and greater than 0.01 are summarized with two asterisks and ‘non-significant (ns)’ respectively.

In both co-culture media, the 16S rRNA gene abundance profile of *F*. *prausnitzii* and *R*. *inulinivorans* followed a similar trend compared to their respective mono-cultures (Fig 3A and 3B). No significant differences were observed between the mono and co-cultures of *F*. *prausnitzii* and *R*. *inulinivorans* in each growth medium. However, comparing the growth profile of each species between two different medium, we observed that rise in the number of 16S rRNA copies of *F*. *prausnitzii* in the co-cultures was significantly higher in the YCFAGD medium than that of the YCGD medium, with reference to the corresponding mono-culture controls (Student t-test, p-value < 0.01), whereas *R*. *inulinivorans* exhibited significant differences in 16S rRNA copies between the mono-cultures and co-cultures of different medium (Student t-test, p-value < 0.01). Glucose and disaccharides were consumed at similar rates between the mono- and co-cultures in the medium with added acetate (S2 Fig). However, the medium without acetate showed a slower consumption of disaccharides in *F*. *prausnitzii* mono-culture and modestly slower consumption of glucose in *R*. *inuliniv*orans mono-culture relative to the co-culture (S2 Fig). In the YCFAGD medium, we observed a relationship between acetate consumption and butyrate formation for all the cultures (Fig 3C–3F). However, in YCGD medium, *F*. *prausnitzii* produced acetate when grown in mono-culture rather than consuming its own secreted acetate in the medium post 8 hrs of fermentation (Fig 3E). For the *F*. *prausnitzii* mono-culture, pH was measured to be 5.5 and 6.5 at 12 h in the YCFAGD and YCGD media, respectively (S2 Fig). The higher pH observed for *F*. *prausnitzii* grown in the YCGD medium can be explained by the lower concentrations of lactate, formate and butyrate compared to the *R*. *inulinivorans* mono-culture in the same medium at a similar time point.

To evaluate whether differences in initial inoculum densities affected relative growth between mono- and co-cultures, both species were co-cultured in YCGD medium, with a higher inoculum density of *R*. *inulinivorans* than *F*. *prausnitzii* (S3 Fig). In brief, we observed that both microbial species grew together in co-culture without inhibiting each other and reached stationary phase upon exhaustion of fermentable substrates. No significant differences were observed between the mono and co-cultures of each species (Student t-test, p-value < 0.01). Since both species shared a similar range of pH optima in correspondence to their similar metabolic product profile in YCFAGD medium, there was no pH-induced growth inhibitory effects as was observed with the first species pair (B. adolescentis and B. thetaiotaomicron). The absence of this or other inhibitory effects observed between *Faecalibacterium prausnitzii* and *Roseburia inulinivorans* in co-culture suggest that these species can generally co-exist without interfering with one another, which is consistent with the positive correlation identified in the co-occurrence network analysis.

## Discussion

In this work, we investigated the co-occurrence patterns between pairs of microbial species with *in vitro* fermentation analyses. Our results support the value of incorporating co-occurrence network analysis into the repertoire of statistical methodologies available to microbial ecologists. Analysis of the co-occurrence network enabled us to identify whether different microbial species that potentially exhibit perturbed growth in co-culture relative to their respective mono-cultures, and these pairs were experimentally investigated with growth and metabolite measurements in mono- and co-cultures. By measuring metabolite concentrations and pH within the different culturing conditions, we observed that the metabolic and environmental changes in the cultures influenced the relative growth of the different species.

Previous studies have investigated the growth of several different strains of genera Bifidobacterium and Bacteroides co-cultured in the presence of different single carbon sources (e.g., glucose, inulin and exopolysaccharides), and generally showed higher population levels of *Bacteroides* than *Bifidobacterium* [36]. Based on all the co-cultures between these two species that have been experimented so far, we have observed a pattern of negative co-occurrence for this pair. This observation aligns well with our finding as predicted from the co-occurrence network analysis. Here, however, we co-cultured the species *Bifidobacterium adolescentis* and *Bacteroides thetaiotaomicron* in a mix of carbon sources such as starch, glucose and maltose, which has not yet been reported. Interestingly, we observed higher population levels of *B*. *adolescentis* than *B*. *thetaiotaomicron* in the co-culture at longer incubation times in comparison to their respective mono-cultures. As proposed earlier, in the case of a negative co-occurrence, the two species could be directly competing with each other, e.g. for nutrients, or they could be so different in their physiological requirements that they never occupy the same niche. Inhibition of the growth of *B*. *thetaiotaomicron* at pH below 5.8 (due to SCFA production) apparently created a niche that could be exploited by low-pH tolerant *B*. *adolescentis*. It is well known that many species from the genus Bacteroides are less acid-tolerant, however, there are a few which can grow at low pH [37,38], hence we avoid generalization of all Bacteroides species to be acid-sensitive. Our results were also consistent with the fact that pH influences the fitness of different microbial species, an effect which is driven by the metabolic activity of different bacteria in a community. Therefore, as pH changes with dietary intake, dynamics in gut bacterial populations are expected to be impacted accordingly [35]. Although one cannot extrapolate from negative correlation to negative interaction with certainty due to unknown mechanisms taking place at the molecular level, we summarize that *Bacteroides thetaiotaomicron* and *Bifidobacterium adolescentis* could not continue to grow together at their optimal level due to SCFA-driven changes in pH of the environment. In addition, we note from previous studies, that combinations of microbial genera at the species or strain level between the gut commensals could possibly be considered as a factor contributing to the variation and coexistence of species in a community.

In a densely populated environment such as the gut, there will be many species that prefer similar conditions and substrate preferences. Nonetheless, this does not necessarily equate to positive correlation, as they would be competing with each other to obtain and meet their similar requirements. This hypothesis led us to investigate the co-occurrence patterns between two species that share similar metabolic pathways and which have not been studied previously in the context of gut bacteria. It has been shown that butyrate producers like *F*. *prausnitzii* and *R*. *inulinivorans* can utilize acetate as a co-substrate from other acetate producing gut bacteria [29,39–41]. They can utilize external acetate to produce butyrate via the butyryl CoA:acetate CoA transferase route [42]. Given that the majority of the cohabitating gut bacteria produce acetate *in vivo*, it is expected that acetate would be found at high concentration in the gut. We therefore explored two media conditions in order to investigate the co-occurrence pattern of these two butyrate-producers in a hypothetical focal area where there is not sufficient acetate. Presence and absence of acetate in the media demonstrated how changes in SCFA metabolism affect the production levels of different SCFAs. Specifically, how *F*. *prausnitzii* shifts between alternative pathways and produces varying proportions of fermentation products (lactate, formate, acetate) depending on the environmental conditions present. In the acetate-free medium, there was decreased butyrate production by *F*. *prausnitzii*, whereas acetate abundance exhibited a smaller effect on the metabolism of *R*. *inulinivorans*. This difference in marginal growth corresponds to a minimal dependence upon acetate for growth and metabolism by *F*. *prausnitzii* [29]. In contrast, *R*. *inulinivorans* appears to produce enough acetate on its own to initiate further conversion of this metabolite to butyrate. In conclusion, we show that both species can coexist with marginal difference from their respective mono-cultures despite different levels of SCFA production and pH variation in YCFAGD and YCGD media.

## Conclusion

Provided the significance of correlation networks, complementation of the *in-silico* predictions from metagenomic data sets with experimentally verified microbial patterns would be invaluable to progress in the area of microbial relationships. Overall, we demonstrated how co-occurrence networks can be used to identify species pairs of interest, and experimentally showed their growth and metabolic dynamics in mono- and co-culture conditions. From the co-occurrence network analysis of abundant bacterial species, it was of great interest to investigate and verify the positive co-occurrence pattern between species (Faecalibacterium and Roseburia) of similar metabolic type as predicted from the network, as they would be expected to compete to meet their similar metabolic requirements. While Bifidobacterium and Bacteroides were predicted to co-occur in a negative fashion even when their metabolic pathways of producing few of the major short chain fatty acids were different from each other. These findings are of value in the design of synthetic microbial communities. Top-down approaches such as this setup will facilitate the selection of bacteria when formulating a community, in order to enable a stable co-existence of multiple bacterial species. Likewise, designing synthetic microbial community with a finite number of species to test the difference in bacterial correlations between the healthy and diseased groups in mouse models may reveal the link between microbial factors and disease susceptibility.

## Supporting information

S1 Fig Depicts the influence of pH on the growth abundance of *Bacteroides thetaiotaomicron* during 24 h of fermentation. A. Log_10_ 16S rRNA gene abundance and pH of *B*. *thetaiotaomicron* during growth with a pH decrease performed at 12.5 h of fermentation, followed by a pH increase at 18.5 h. B. Log_10_ 16S rRNA gene abundance and pH of *B*. *thetaiotaomicron* during growth with a pH increase initiated at 12.5 h of fermentation, followed by a decrease performed at 18.5 h of fermentation. Experiments were performed in duplicates. The values represent the mean of each biological replicate.
(TIFF)

S2 Fig Extracellular metabolite concentration of *Faecalibacterium prausnitzii* and *Roseburia inulinivorans* grown in YCFAGD (left panels) and YCGD (right panels) medium. A-B. Glucose and C-D. Disaccharide consumption, E-F. Lactate and G-H. Formate production and I-J. pH profile of *F*. *prausnitzii* and *R*. *inulinivorans* mono- and co-cultures in YCFAGD and YCGD medium for 50 h. FR = co-culture of *Faecalibacterium prausnitzii* and *Roseburia inulinivorans*, Fpr = mono-culture of *F*. *prausnitzii*, Rin = mono-culture of *R*. *inulinivorans*. Experiments were performed in triplicates and error bars represent the standard deviation between each biological replicate.
(TIFF)

S3 Fig Log_10_ 16S rRNA copies of *Faecalibacterium prausnitzii* and *Roseburia inulinivorans* in mono- and co-cultures of YCGD (Yeast Casitone glucose disaccharide) medium for 40h. Experiments were performed in triplicates and error bars represent the standard deviation between each biological replicate. P-values less than and greater than 0.01 are summarized with two asterisks and ‘non-significant (ns)**’** respectively.
(TIFF)
