# Supplementary figures and images for: In vitro co-cultures of human gut bacterial species as predicted from co-occurrence network analysis
Source: PLoS One. 2018 Mar 30;13(3):e0195161. doi: 10.1371/journal.pone.0195161 (PMC5877883; doi:10.1371/journal.pone.0195161)

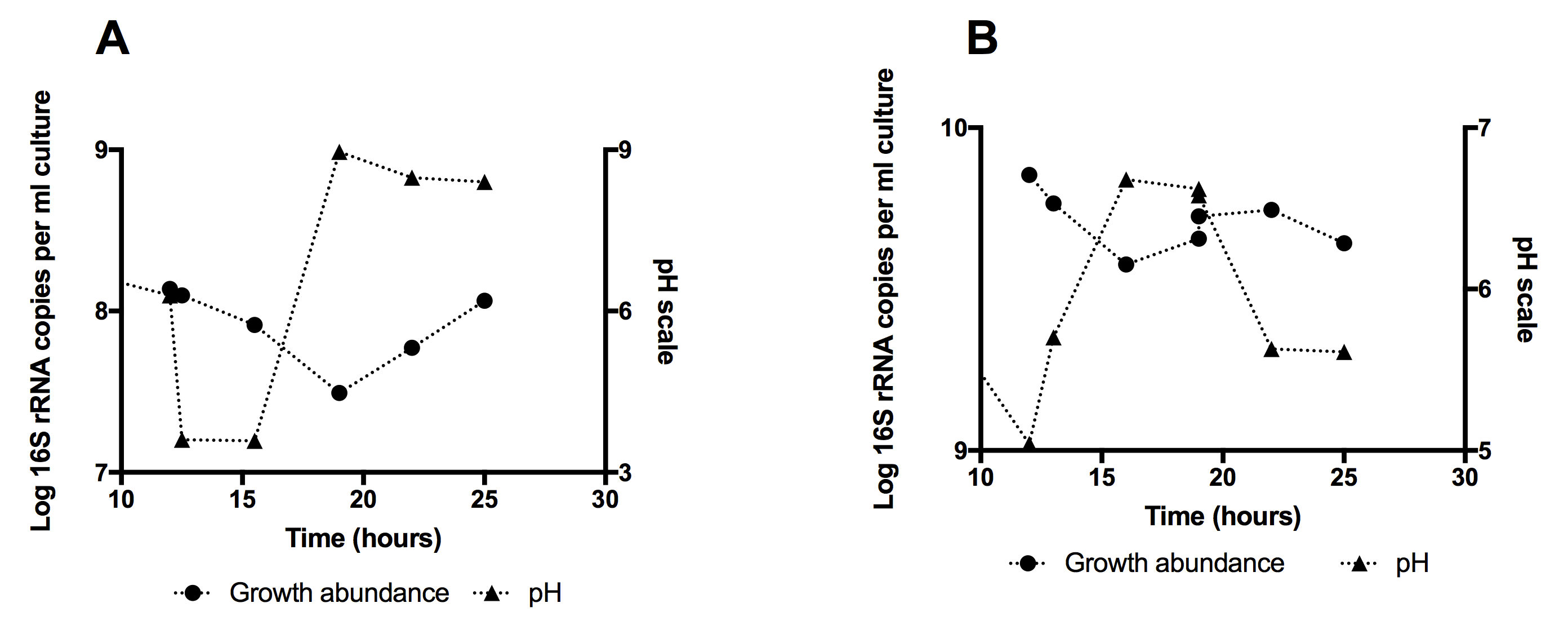

Supplement: S1 Fig — A. Log10 16S rRNA gene abundance and pH of B. thetaiotaomicron during growth with a pH decrease performed at 12.5 h of fermentation, followed by a pH increase at 18.5 h. B. Log10 16S rRNA gene abundance and pH of B. thetaiotaomicron during growth with a pH increase initiated at 12.5 h of fermentation, followed by a decrease performed at 18.5 h of fermentation. Experiments were performed in duplicates. The values represent the mean of each biological replicate. (TIFF) [file pone.0195161.s001.tiff]

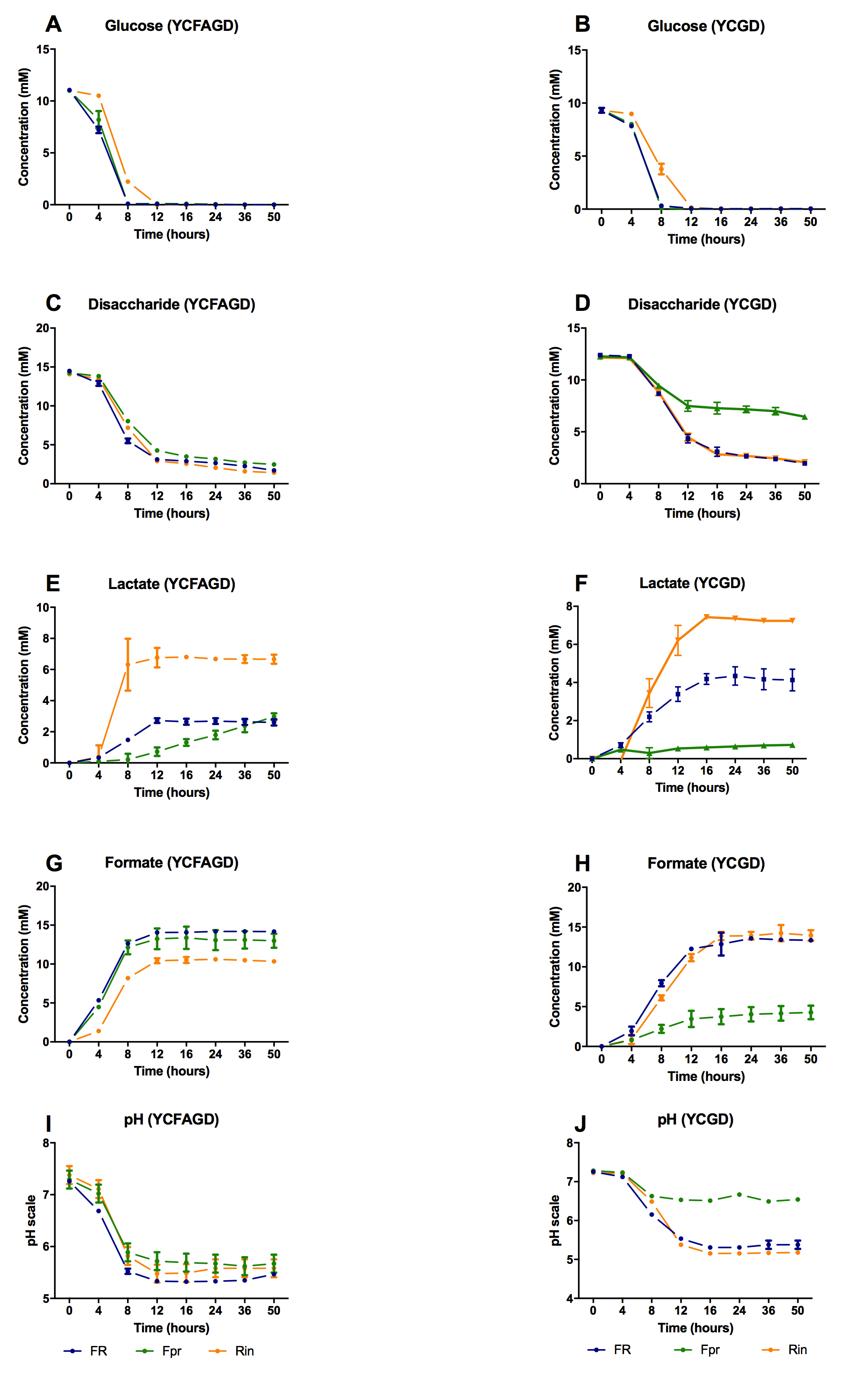

Supplement: S2 Fig — A-B. Glucose and C-D. Disaccharide consumption, E-F. Lactate and G-H. Formate production and I-J. pH profile of F. prausnitzii and R. inulinivorans mono- and co-cultures in YCFAGD and YCGD medium for 50 h. FR = co-culture of Faecalibacterium prausnitzii and Roseburia inulinivorans, Fpr = mono-culture of F. prausnitzii, Rin = mono-culture of R. inulinivorans. Experiments were performed in triplicates and error bars represent the standard deviation between each biological replicate. (TIFF) [file pone.0195161.s002.tiff]

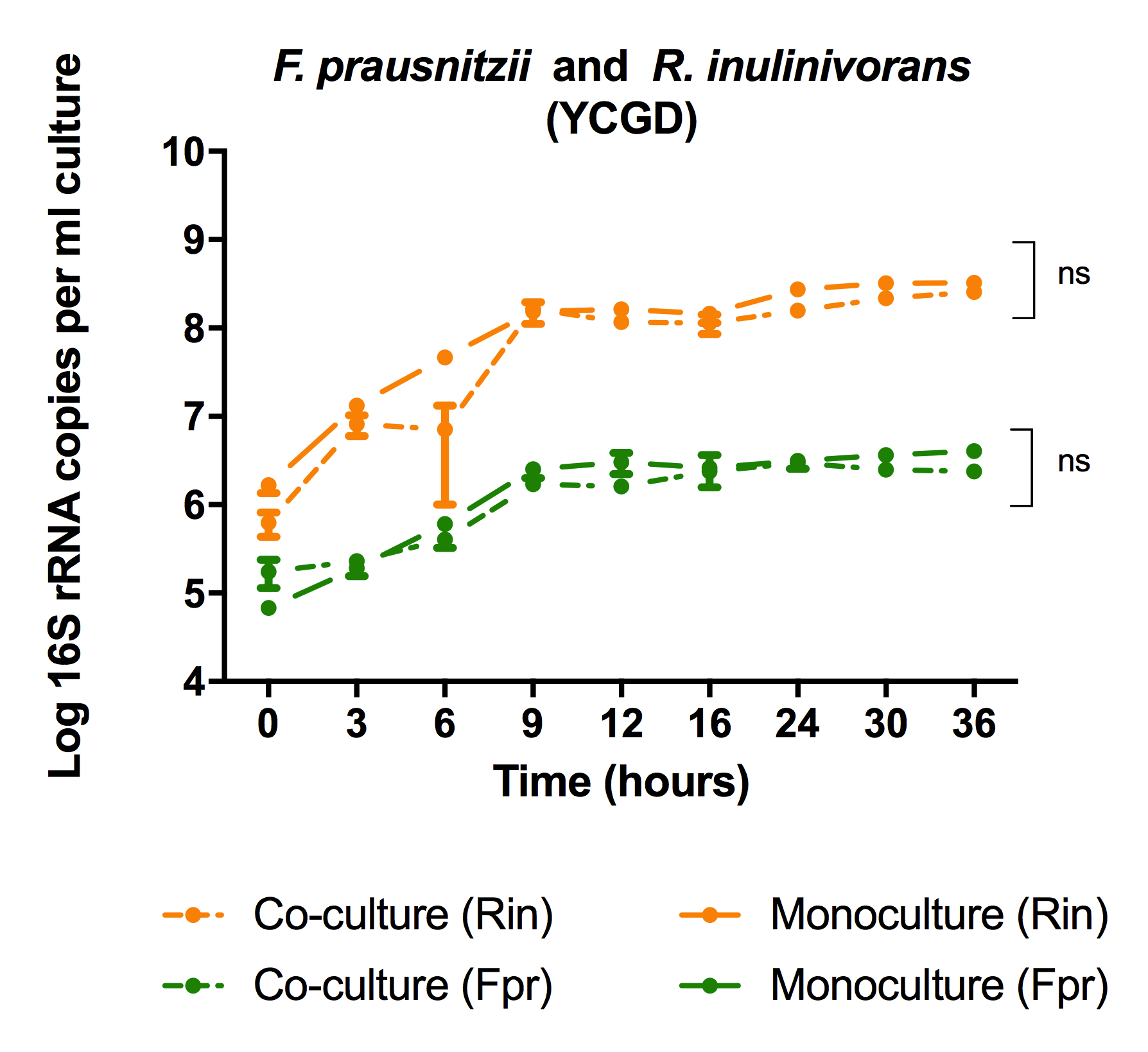

Supplement: S3 Fig — Experiments were performed in triplicates and error bars represent the standard deviation between each biological replicate. P-values less than and greater than 0.01 are summarized with two asterisks and ‘non-significant (ns)’ respectively. (TIFF) [file pone.0195161.s003.tiff]
